# Tegumentary leishmaniasis mimicking visceralization in a cirrhotic patient: atypical cutaneous lesions and local immunological features

**DOI:** 10.1590/0037-8682-0380-2019

**Published:** 2020-01-27

**Authors:** Sebastian Vernal, Yuri Casal, Lucas T. Vieira, Valdir S. Amato, María Irma S. Duarte, Ana Catharina S.S. Nastri

**Affiliations:** 1 Universidade de São Paulo, Departamento de Doenças Infecciosas e Parasitárias, São Paulo, SP, Brasil.; 2 Universidade de São Paulo, Departamento de Patologia, São Paulo, SP, Brasil.

**Keywords:** Leishmaniasis, Cutaneous Leishmaniasis, Visceral Leishmaniasis

## Abstract

Tegumentary leishmaniasis (TL) diagnosis is challenging due to the lack of a gold standard diagnostic tool. The diagnosis is significantly harder in regions where visceral leishmaniasis (VL) is also prevalent since immunological tests may present cross-reactivity. A cirrhotic patient from an endemic Brazilian region for TL and VL presented with atypical cutaneous lesions, a usual clinico-laboratory feature of VL (including a positive rk39 test result), but he was diagnosed with TL histopathologically; VL was ruled out by necropsy. Physicians working in co-prevalent areas should be aware of atypical features, unusual clinical course, and unexpected laboratory findings of leishmaniasis.

## INTRODUCTION

Diagnosing American tegumentary leishmaniasis (ATL) is still a difficult task for physicians, even for dermatologists and infectious diseases specialists^1^. Although the diagnosis can occasionally be only based on the clinical-epidemiological criteria, laboratory tests are also important. The lack of a gold standard diagnostic tool prevents the establishment of ATL diagnosis^2^. This could be significantly harder in endemic co-prevalent regions experiencing ATL and visceral leishmaniasis (VL) since immunological tests may present cross-reactivity^3^.

Here, we present a cirrhotic patient from an endemic Brazilian region for both ATL and VL^4^ ^,^ ^5^ who presented with atypical cutaneous lesions, a usual clinico-laboratory feature of VL (including positive results on rk39 test using a bone marrow sample), but the patient was diagnosed with ATL on histopathological analysis; VL was ruled out by necropsy.

## CASE REPORT

**FIGURE 1:**
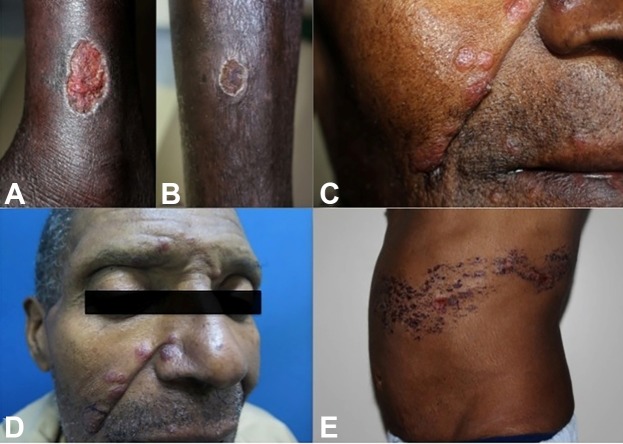
(A) Left leg lesion: well-defined painless ulcer with elevated borders. (B) Right leg lesion: well-defined painless ulcer with peripheral signs of venous chronic insufficiency. (C) Papulonodular *sarcoid-like* infiltrated lesions. (D) Papules in the facial region following a lymphatic trajectory from the glabella to the right perioral region. (E) Multiple painful bullous lesions in the left dorsothoracic region (T5-T7 left dermatome).

In May 2018, a 64-year-old man in *São Paulo*, Southeastern Brazil, with a history of essential hypertension and hepatic cirrhosis due to chronic alcoholism (CHILD B, MELD 18), sought care for weight loss (from 97 kg to 65 kg), asthenia, and episodes of fever for 1 year. Six months earlier, he detected single skin lesions on his left leg (Figure 1A), followed by the appearance of another cutaneous lesion on his right leg (Figure 1B). Three months later, he observed multiple lesions at the glabella, right nose alae, right dimple, and right perioral region (Figure 1C).

He initially visited a dermatologic outpatient clinic. The leg lesion started as a single papule lesion evolving into a well-defined painless ulcer with elevated borders (Figure 1A and Figure 1B). Due to the symmetry of the lesions and the peripheral signs of venous chronic insufficiency, they were considered as venous stasis-related skin ulcers and were not biopsied. Conversely, face skin lesions were described as papulonodular *sarcoid-like* infiltrated lesions (Figure 1C) being biopsied with the suspicion of sarcoidosis, secondary syphilis, and lepromatous leprosy. Physical examination also revealed painless palpable liver and spleen. Blood samples and abdominal ultrasound were requested with brief outpatient return.

In June 2018, patient’s laboratory results revealed pancytopenia (hemoglobin level, 10.4 g/dL; leucocyte count, 3,210/mm^3^ [normal differential]; and platelet count, 108,000/mm^3^), normal renal functions, hyponatremia (sodium level, 127 meq/L), normal potassium level, normal transaminase level, elevated canalicular enzymes (alkaline phosphatase level, 135 g/dL; gamma-glutamyl transferase level, 128 g/dL), elevated total bilirubin, 2.18 g/dL (direct bilirubin level, 1.51 g/dL), normal lipase level, extended prothrombin time (international normalized ratio, 1.58), hypoalbuminemia (albumin, 2.7 g/dL), and normal alpha-fetoprotein level (1.8 g/dL). Serological test revealed negative results for anti-human immunodeficiency virus and anti-hepatitis C virus, nonreactive Hepatitis B virus surface antigen (AgHBs) and antibody (anti-HBs), and reactive antibody to Hepatitis B virus core antigen (anti-HBc) (further polymerase chain reaction [PCR] for hepatitis B virus was undetected);. Further, he was tested positive on treponemic test with Venereal Disease Research Laboratory test 1:2, indirect immunofluorescence (IFI) for leishmaniasis (1:80), and enzyme-linked immunosorbent assay for leishmaniasis (>1:1,280). Ultrasound revealed signs of chronic liver disease with portal hypertension and a significant splenomegaly; no focal hepatic lesions were observed. The histopathology of the facial cutaneous lesion revealed the following: (I) skin with the epidermis presenting hyperparasqueratosis, focal hypogranulosis, irregular acanthosis, discrete spongiosis, and vacuolar degeneration of the basal layer, (II) granuloma formation, and (III) intense lymphohistiocytic infiltration with plasma cells and epithelioid histiocytes. Further tests, including immunohistochemistry, were ongoing at that time.

Fifteen days before the outpatient return, he observed multiple painful bullous lesions in the left dorsothoracic region (Figure 1E), and he initially received (in external service) acyclovir for herpes zoster diagnosis. When examined, the patient still had active disseminated bullous lesions in more than one dermatome; hence, an isolated ward was requested. Regarding the patient’s epidemiology, coming from Araçatuba (western *São Paulo* State, Southeastern Brazil), an endemic region for VL with intense transmission of canine and human cases, and with the patient presenting with weight loss, fever, pancytopenia, hepatosplenomegaly, and elevated titers of leishmaniasis in serological test, the diagnosis of VL was suspected.

Considering the suspicion for VL, a bone marrow sample was collected. Global hypocellularity, appropriate cell maturation with normal morphology, and absence of microorganism or foreign bodies were observed. Bacterioscopy and bacterial/fungi cultures were negative. The rk39 test was positive, and the VL case was reported to the surveillance. After normal electrocardiogram and echocardiogram, 3 mg per kilogram of amphotericin B liposomal (200 mg per day) was initiated.

Unfortunately, during the 9th day of treatment, the patient presented with severe acute respiratory syndrome, refractive shock, and multi-organ failure, resulting to death. Necropsy revealed several hemorrhagic spots in various organs consistent with acute hepatic failure, including an extensive alveolar hemorrhage. Regarding VL diagnosis, histopathological analysis of the liver, spleen, and bone marrow did not reveal any histological pattern suggestive of VL, amastigote forms were not observed, and immunohistochemistry for leishmaniasis was negative.

**FIGURE 2:**
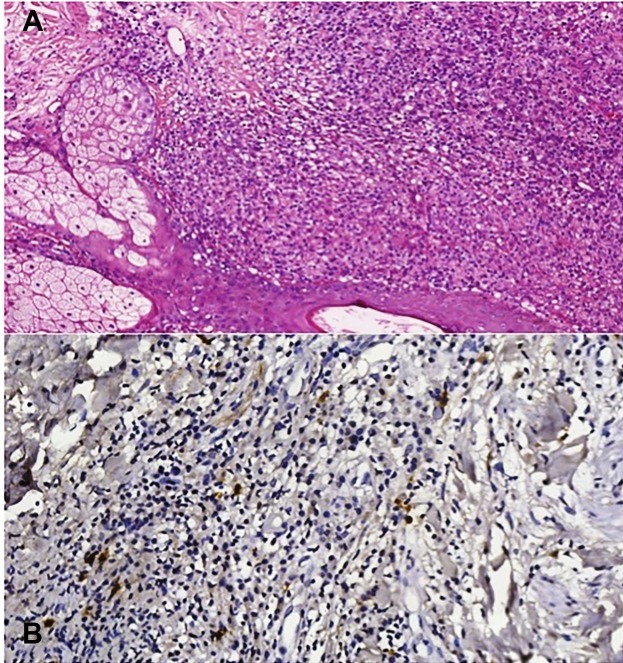
(A) Hematoxylin and eosin staining (40X) showing pseudogranuloma formation with visualization of amastigote forms. (B) Positive immunohistochemistry for leishmaniasis (100X).

**FIGURE 3:**
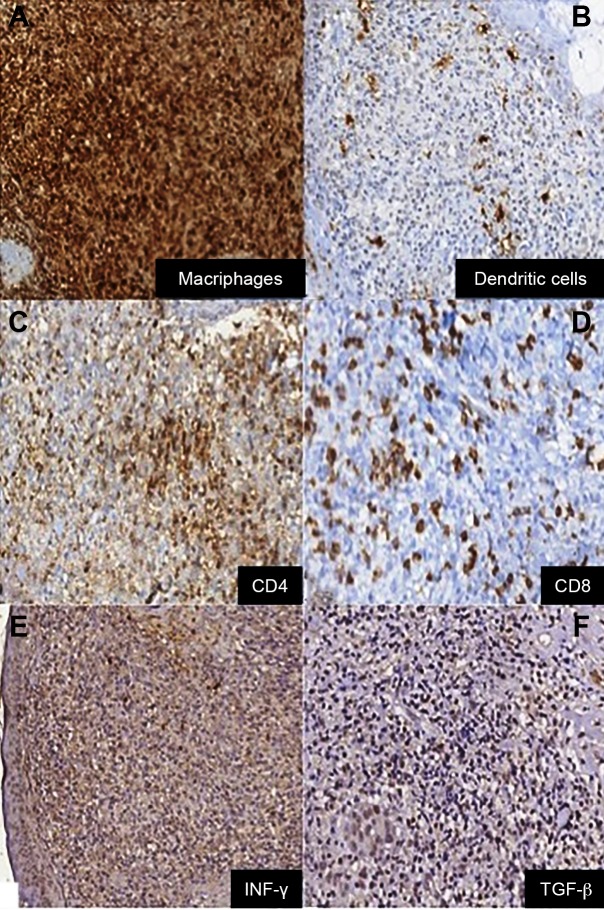
Positive immunohistochemistry (100X) for **(A)** anti-cluster of differentiation (CD) 68; **(B)** anti-S100; **(C)** anti-CD4; **(D)** anti-CD8; **(E)** anti-interferon γ; **(F)** antitumor growth factor ß.

A skin sample of the facial lesions, collected in May 2018, was reanalyzed by a leishmaniasis specialist, which confirmed the presence of amastigote and positive immunohistochemistry for leishmaniasis (Figure 2A and Figure 2B). Immunological local analysis revealed a deficient innate response without the presence of complement (C3=+0/+3) and weak presence of natural killer cells (anti-CD57=+1/+3). Macrophages and dendritic cells were widely distributed in the skin sample (anti-cluster of differentiation [CD] 68 and anti-S100=+3/+3) (Figure 3A and Figure 3B), but with an inappropriate cytokine production (antitumor necrosis factor (TNF] α=+2/+3, anti-interleukin [IL] ß, anti-IL6, and anti-IL8=+1/+3). A suitable adaptive response was observed (anti-CD4 and anti-CD8=+3/+3) (Figure 3C and Figure 3D) modulated to the Th1 pole (anti-interferon γ and anti-TGF ß=+3/+3) (Figure 3E and Figure 3F). The regulatory response was defective with low display of cytokines (anti-CD20, anti-IL17, anti-IL10, and anti-IL4=+1/+3). Deoxyribonucleic acid (DNA) extraction was performed from the biopsy sample, followed by leishmania kDNA detection by PCR; however, the identification of the *Leishmania* sp. (paraffin tissue sample with poor-quality DNA extraction) was not possible.

## DISCUSSION

This case report offers some interesting and controversial data: (a) an unusual clinical feature of ATL with atypical infiltrated *sarcoid-like* papules in the facial region following a lymphatic trajectory (Figure 1D) from the glabella to the right perioral region. We cannot consider the leg ulcers as the primary local ATL infection since they were not biopsied and they were initially considered as vascular stasis-related lesions, emphasizing the difficulty in diagnosing ATL, even for experienced specialists^1^. (b) Immunological local features indicated a deficiency in innate response, but a strong Th1 response with an intense pro-inflammatory activity. Furthermore, the lack of regulatory response leads to severe local damage, which explains the atypical skin lesion. Regulation deficiency may be explained not only by the immunosuppressed patient conditions (chronic hepatopathy) but also by *Leishmania* sp*.* local immunomodulation^6^ ^,^ ^7^. On the contrary, parasite exposure and a deficient cellular activity lead to an intense humoral response with high titers of anti-Leishmania circulating antibodies, confirmed by great titers of IFI and ELISA. (c) Following the Brazilian Ministry of Health VL guidelines^4^, this patient met the criteria to confirm the diagnosis of VL; however, with the patient’s comorbidities (cirrhosis with portal hypertension) and ATL, the clinico-laboratory interpretation was significantly harder. Moreover, rK39 false-positive test supported the misdiagnosis, ruling out postmortem VL. The rK39 test is a valuable tool for VL diagnosis^8^, showing a good accuracy depending on the test brand and the global region used^9^. However, some rK39 rapid test brand had shown excellent performance in the blood from Brazilian patients^10^; its results should be carefully analyzed in co-prevalent regions with ATL due to an eventual cross-reactivity^3^, as observed here, even in bone marrow samples. Regarding surveillance, VL notification had to be removed. (d) Otherwise, considering the patient’s immunosuppression and the high incidence of VL in Southeastern Brazil, these atypical lesions may also be explained due to the cutaneous involvement by viscerotropic *Leishmania* strains^11^.

Physicians working in co-prevalent areas experiencing ATL and VL should be aware of the atypical features, unusual clinical course, and unexpected laboratory findings of leishmaniasis, determining the possibility of leishmaniasis coinfection and/or cross-reactivity among diagnostic exams. Additionally, patients’ comorbidities attributing to VL findings mimicking baseline pathologies should be carefully studied.
